# Fluctuation analysis in the dynamic characteristics of continental glacier based on Full-Stokes model

**DOI:** 10.1038/s41598-019-56864-3

**Published:** 2019-12-27

**Authors:** Zhen Wu, Huiwen Zhang, Shiyin Liu, Dong Ren, Xuejian Bai, Zhaojie Xun, Zhentao Ma

**Affiliations:** 1 Lanzhou Geophysical National Field Scientific Observation and Research Station, Earthquake Administration, Earthquake Administration of Gansu Province, Lanzhou, 730000 China; 2 State Key Laboratory Breeding Base of Desertification and Aeolian Sand Disaster Combating, Gansu Desert Control Research Institute, Lanzhou, 730070 China; 3 Institute of International Rivers and Eco-Security, Yunnan University, Kunming, 650091 China

**Keywords:** Cryospheric science, Environmental impact

## Abstract

Ice thickness has a great influence on glacial movement and ablation. Over the course of the change in thickness, area and external climate, the dynamic process of how glaciers change and whether a glacier’s changes in melting tend to be stable or irregular is a problem that needs to be studied in depth. In our study, the changes in the dynamic process of the No. 8 Glacier in Hei Valley (H8) under the conditions of different thicknesses in 1969 and 2009 were simulated based on the Full-Stokes code Elmer/Ice (http://www.csc.fi/elmer/). The results were as follows: (1) The thickness reduction in glaciers would lead to a decrease in ice surface tension and basal pressure and friction at the bottom, and the resulting extensional and compressional flow played an important role in the variations in glacial velocity. (2) The force at the bottom of the glacier was key to maintaining the overall stress balance, and the glaciers that often melted and collapsed in bedrock were more easily destroyed by the overall force balance and increased change rate of glacial thaw. (3) Temperature changes at different altitudes affected the ice viscous force. The closer the ice surface temperature was to the melting point, the greater the influence of temperature changes on the ice viscous force and ice surface velocity. Finally, we used the RCP 4.8 and 8.5 climate models to simulate the changes in H8 over the next 40 years. The results showed that with some decreases in ice surface compression and tension, the gravity component changes caused by local topography begin to control the ice flow movement on the surface of glacier, and melting of the glacial surface will appear as an irregular change. The simulation results further confirmed that the fluctuation in glacial dynamic characteristics could be attributed to the change in the gravity component caused by ablation.

## Introduction

Understanding the potential impacts of climate on glaciers is significant in studying regional climate, water resources and predictions of future glacial changes^1–4^. Therefore, researchers have observed many of the glacial changes in the Tianshan Mountains, including glacial area change^5–8^, volume change^9–12^, variation in mass balance^7,13–15^, runoff characteristics^16–19^ and so on. These studies reflect the changes in some factors associated with glacial melting; however, these studies do not clearly explain the state and potential trends of glaciers based on the mechanism of glacial movement. Because glacial dynamic characteristics exert an imperceptible influence on glacial processes, there is more contingency and complexity in the process. That is, glacial stress changes with temperature and thickness all the time, including changes in velocity, glacial melting and hydrological process caused by stress. However, changes in stress are difficult to observe directly. Obviously simple observations can no longer meet the needs of research.

The force exerted on the ice body has a vital influence on the velocity and ablation of glaciers^20–24^. Much of the negative mass balance is the result of climate change, but the creep of the glacier will also lengthen the ice flow and reduce the glacier’s thickness. Therefore, the dynamic process also affects the variation in mass balance to some extent^25^. Understanding the ice deformation mechanism of the main body of glaciers and its effect on glacial flow behaviour is the core of understanding the deformation characteristics of ice body units^26^. Deformation rates largely depend on extrinsic parameters, such as atmospheric temperature, surface pressure caused by precipitation or snowfall, and water content caused by ice ablation^27,28^. Moreover, the ice deformation rate will govern the overall flow and ablation behaviour of glaciers. Together, plastic deformation and stress lead to distortion and deformation of ice^29,30^.

To simulate and analyse the relevant parameters, we used the Elmer/ice model in this study. Although there have been similar glacial dynamic simulation studies before, only a few researchers have performed glacial research using the two-dimensional ice flow model in the Tianshan Mountains^31^, and there is a great lack of complex force analysis available for this area. In this study, we employed the incompressible fluid, momentum and continuity equations to simulate the glacial stress variation under conditions of two types of glacial morphology and analysed the reason for ice velocity and stress changes. Compared with previous studies, we adjusted the viscous parameter *A*, and very few adjustments were made to other environmental settings except glacial topography and boundary conditions. The diagnostic simulation presented the differences in stress on the surfaces and bottoms of glaciers in 1969 and 2009. The influences of both external and internal factors on glacial movement and ablation were analysed simultaneously. Finally, future change trends of glaciers was also discussed, including the effects of dynamic characteristics on ice flow velocity, force, englacial structure and hydrological processes. Our prognostic simulations verified the results of previous studies and future trends are presented.

**Figure 1:**
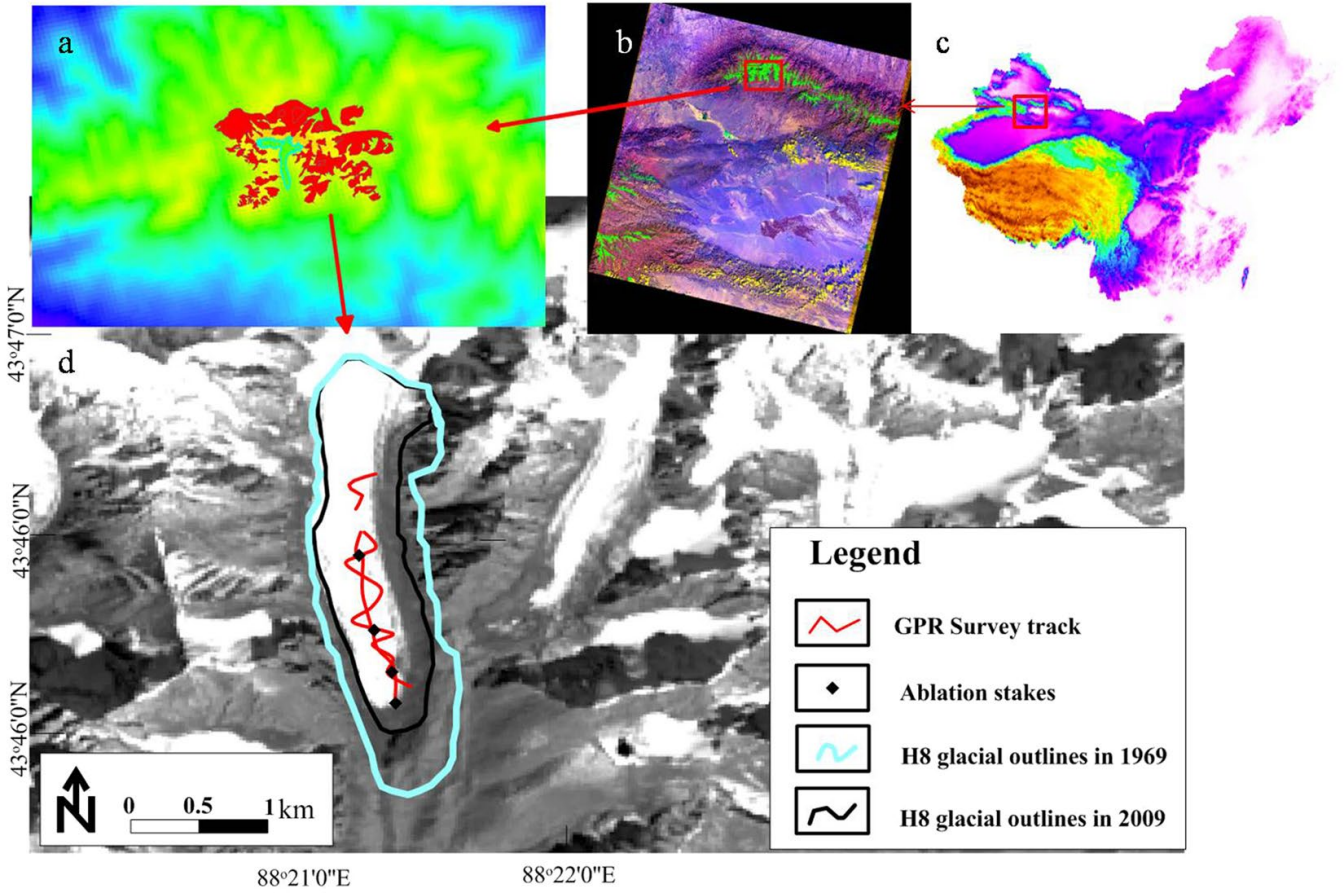
Overview of the study sites and GPR track. (**a**) The overall outline of the H8 and the map was plotted using ArcGIS 10.2 (http://www.esri.com/). (**b**) The Landsat image of glacial location (https://glovis.usgs.gov/). (**c**) Digital Elevation Model (DEM) in Mainland China. (**d**) The glacial outlines in 1969 and 2009 extracted separately from Chinese Military Geodetic Service maps and Landsat image (https://glovis.usgs.gov/). The map was plotted using ArcGIS 10.2 (http://www.esri.com/) and Surfer 16 (https://www.goldensoftware.com/).

The No. 8 glacier in the Hei Valley (H8) (43°44′–43°47′N, 88°19′–88°22′S) (Fig. 1) is located on the southern slope of the Bogda Peak in the Tianshan Mountains. With a terminal point of 3390 m, H8 is approximately 5000 m at its highest point, and has an area of 5.7 km2. The climate in this area is a typical continental climate with less precipitation, which is concentrated in summer^32^.

## Method Acquisition

The numerical solutions of the Full-Stokes equations were obtained using the finite element method based on the code Elmer (http://www.csc.fi/elmer). The Elmer software was developed in Center for Science (CSC) Ltd., Espoo, Finland, with worldwide collaborators. Considering all stress components, Elmer can solve the thermomechanically coupled problem, such as the Full-Stokes problem of flow^20,33–35^. The momentum and continuity equations and incompressible heat equations have been used to describe the ice velocity, temperature and stress. The specific equation descriptions are as follows.

### The expression of velocity

Glaciers belong to incompressible flow, so the incompressible flow equation in the Stokes model can be used. The equation is as follows:

1 $$\nabla \cdot u=0$$

2 $$\rho (\frac{\partial u}{\partial t}+(u\cdot \nabla )u)-\nabla \cdot (2\mu \varepsilon )+\nabla p=\rho f$$

where $\nabla u$ is the curl of a velocity field, *ρ* is the density of ice, *g* is gravity, and *p* is pressure. *ε* is a linearized strain rate tensor that can be written as follows:

3 $${\varepsilon }_{ij}=\frac{1}{2}(\frac{\partial {u}_{i}}{\partial {x}_{i}}+\frac{\partial {u}_{j}}{\partial {x}_{i}})$$

The viscosity *η* is described as follows:

4 $$\eta =\frac{1}{2}{(EA(T))}^{-1/n}{d}^{(1-n/n)}$$

where $d=\sqrt{\frac{1}{2}tr({D}^{2})}$ is the effective strain rate, $D=1/2(gradv+grad{v}^{T})$ represents the strain tensor, and *T* is a parameter describing the pressure melting.

### The boundary conditions of velocity and stress

Because of the complexity of bottom sliding in continental glaciers, basal sliding also contributes to ice motion. The Dirichlet boundary condition for the velocity component is applied for basal sliding, and no sliding at the bottom is set as follows:

5 $${u}_{b}=0$$

Under conditions of a stress-free glacial surface, the atmospheric temperature is taken as a function of the elevation relationship.

Because there is no flow across the surface of the glacier, the following equation is applicable:

6 $$\overrightarrow{u}\cdot \overrightarrow{n}=0$$

where $\overrightarrow{n}$ is the outward unit normal to the boundary.

Surface stress can be divided into normal stress and tangential stress. Normal stress is usually repressed in the following form:

7 $${\sigma }_{n}=\frac{\gamma }{R}-{p}_{a}$$

where *γ* is the surface tension coefficient, *R* is the mean curvature and *p*_*a*_ is the atmospheric pressure. The expression of tangential stress is as follows:

8 $$\overrightarrow{\sigma }={\nabla }_{s}\gamma $$

where ${\nabla }_{s}$ is the surface gradient operator.

The coefficient *γ* is a thermophysical property depending on the temperature. Temperature differences on the surface influence the transport of momentum and heat near the surface. This phenomenon is called Marangoni convection or thermocapillary convection. The temperature dependence of the surface tension coefficient can be approximately expressed by a linear relation. To facilitate the calculation, the value of *γ* was set to 0.5 in this study.

### The expression of ice temperature

Because the temperature of ice affects the viscoplasticity of the ice, it is necessary to iterate the force equation and temperature equation together. The incompressible heat equation is expressed as follows:

9 $$\rho c(\frac{\partial T}{\partial t}+(\overrightarrow{u}\cdot \nabla )T)-\nabla \cdot (k\nabla T)=\overline{\overline{\tau }}:\overline{\overline{\varepsilon }}+\rho h$$

where ρ is the density, *c*_*p*_ is the heat capacity at constant pressure, *T* is the temperature, $\overrightarrow{u}$ is the convection velocity, *k* is the heat conductivity and *h* is the source of heat. The term $\overline{\overline{\tau }}:\overline{\overline{\varepsilon }}$ is the frictional viscous heating, which can be ignored in most cases. This setting was also ignored in our study. In addition, we set $\overrightarrow{u}$ = 0. For solids, conductivity may be anisotropic, and the conductivity is a tensor.

Phase change modelling is performed by modifying the definition of heat capacity according to whether a point in space is in a solid phase, liquid phase or in a “mushy” region. The choice of heat capacity within the intervals is explained in detail below. When the phase change occurs within a finite temperature range, enthalpy is needed when the solidification phase change model Elmer is used. The enthalpy is defined as follows:

10 $$H(T)={\int }_{0}^{T}(\rho {c}_{p}+\rho L\frac{\partial f}{\partial \lambda })d\lambda $$

where *f*(*T*) is the fraction of liquid material as a function of temperature, and *L* is the latent heat. When using the enthalpy-temperature curve to compute an effective heat capacity, Eq. 10 is identical to the heat equation.

### Boundary condition of ice temperature

For temperature, we can apply boundary conditions and specify temperature or heat flux. In the Dirichlet boundary condition (temperature is prescribed), the heat flux depends on the heat transfer coefficient *α* and the external temperature, which can be written as follows:

11 $$-k\frac{\partial T}{\partial n}=\alpha (T-{T}_{ext})$$

where *T*_*ext*_ can be input as the geothermal and external atmospheric temperatures.

The surface temperature is affected by the temperature gradient that varies with altitude. The subglacial boundary condition of the temperature simulation is the mean annual atmospheric temperature, while the bedrock boundary condition is a prescribed geothermal heat flux gradient. The formula is as follows:

12 $$\frac{\partial {T}_{b}}{\partial z}=-\frac{G+{\tau }_{b}{u}_{b}}{{k}_{1}}$$

We set $G=-{k}_{r}(\partial T/\partial z)$, which is similar to Pattyn^36^. The compilation of heat flow data in China^37^ shows that the geothermal flux value near the glacier ranges from 40 to 50 mWm^−2^. Therefore, the geothermal flux value at the bedrock location was set to an average value (45 mWm^−2^) in our study.

Due to the lack of relevant measured data, especially data on ice temperature and subglacial hydrological process data, it is difficult to determine the parameters that are more suitable for the study area. Therefore, most of the parameters are set to the most commonly used values. The specific parameter values are shown in Table 1.

**Table 1:** The value of some parameters in the model.

Symbol	Parameter	Value	Unit
*σ*	internal stress	calculate	Nm^−2^
*F*_*latt*_	width factor	calculate	nothing
*T*	effective stress	calculate	Nm^−2^
*A*	function of ice temperature	2.5 × 10^−24^	Pa^−3^ s^−1^
*ρ*	ice density	917	kgm^−3^
g	gravitational acceleration	9.8	ms^−2^
*R*	universal gas constant	8.31424	Jmol^−1^K^−1^
*T*_*m*_	melting temperature of ice at atmospheric pressure	273.15	K
*β*	pressure melting point coefficient	9.7 × 10^−4^	km^−1^
*A*_0_	temperature-independent flow-law coefficient	8.75 × 10^−13^	Pa^−3^s^−1^
*n*	flow-law exponent	3	nothing
*G*	geothermal flux	45	mWm^−2^
*k*_1_	thermal conductivity of ice	2.1	Wm^−1^ K^−1^
*u*_*b*_	ice flow velocity at the bedrock position	0	ma^−1^
*N*	basal effective pressure	10^−30^	yr^−1^
*C*	coulomb friction law constant	3	nothing
*C*_*p*_	heat capacity	2050 (−10 °C)	jkg^−1^ C^−1^
_*u*_	x direction heat advection velocity	calculate	ms^−1^
*ω*	y direction heat advection velocity	calculate	ms^−1^
*A*_*b*_	sliding constant	0.7	nothing
*v*	viscosity	calculate	nothing
*θ*_*pmp*_	melting point temperature	−2	°C
*ψ*_*w*_	subglacial water flux	0.5	m^3^ m^−2^

### The method of thickness acquisition for 1969 and 2009

In September 2009, ice thickness of the H8 glacier was investigated using a ground penetrating radar (GPR) (Pulse EKKO-PRO, Sensors & Software Inc., Canada). Simultaneously, a global positioning system (GPS) device (GPS76, Beijing UniStrong Science & Technology, China) was used to position each profile and obtain surface elevation in 2009. At the same time, our study team also obtained some surface elevation points outside the GPR survey line. All GPS data were presented in the China Geodetic Coordinate System 2000 (CGCS2000) format. To compare these data with the surface elevation data in 1969, we used the Kriging method to interpolate all the data obtained by GPR and GPS into a contour interval of 5 m and generated a digital elevation model (DEM) in 2009 with a pixel resolution of 30 m.

**Figure 2:**
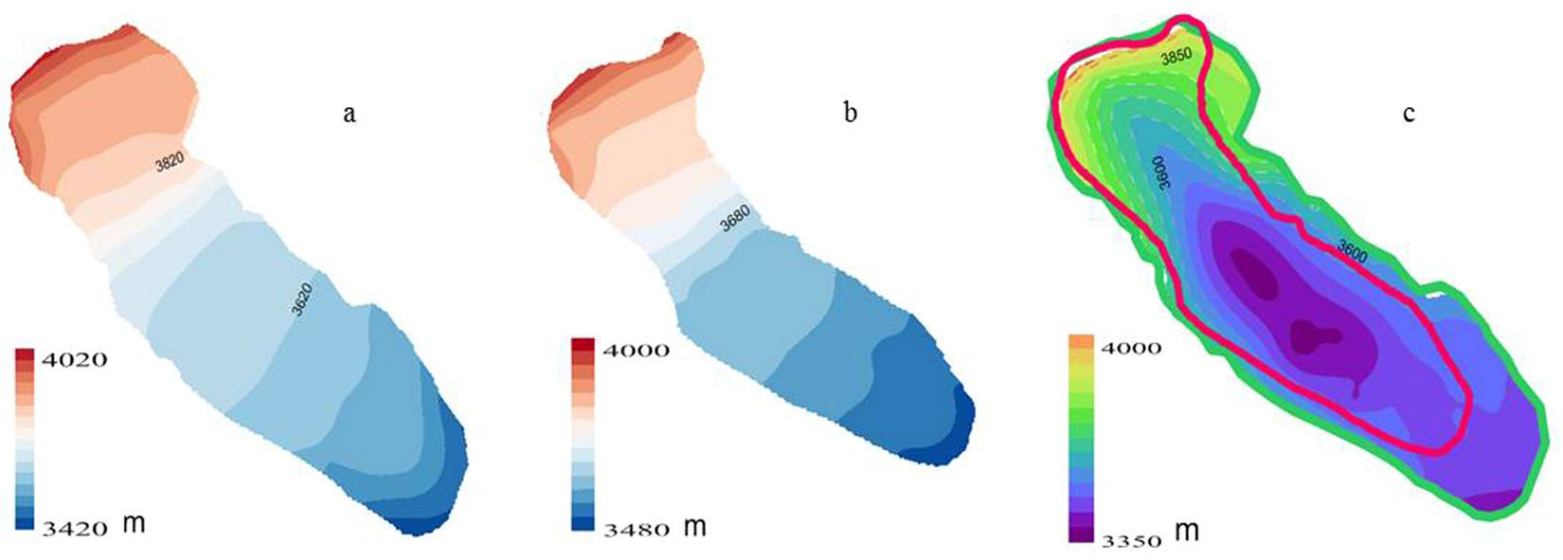
The surface and subglacial terrain of H8. (**a**) The surface terrain of H8 in 1969; (**b**) the surface terrain of H8 in 2009; (**c**) the bottom topography of H8.

In our study, we used a digital topographic map of China of 1:50000 in 1969 measured by the Chinese Military Geodetic services. The topographic map was first scanned at 600 dpi, and then, the 20 m and 5 m contour intervals and determined point height were digitized to produce DEMs in 1969 with a 30 m cell size (Fig. 2a). The coordinates of the DEMs were expressed in the Beijing 54 Krasovsky coordinate system. The flatness of Beijing 54 is different from that of CGCS 2000. To unify their coordinates, we used five national trigonometric reference points of Beijing 54 and CGCS 2000 to calculate the conversion parameters. All the data on the topographic map were reprojected and transferred into CGCS 2000.

To evaluate the error, we randomly selected five nonglacialized areas below 4500 m.a.s.l., all of which had slopes of less than 15°. The results showed that the average error was 6.3 m, which was equivalent to advanced spaceborne thermal emission and reflection radiometer (ASTER) data (6.24 m)^38^. The magnitude of ice thickness fluctuation had little effect on the simulation results based on the ice thickness of the two phases, so we ignored the effect of the error caused by the transformed DEM data.

The GPR data in 2009 were used to obtain the ice thickness data. We supposed that the bedrock elevation was changeless. The ice thickness in 1969 was provided by the DEM in 1969 minus the bedrock terrain in 2009. The surface and subglacial terrain of H8 is shown in Fig. 2b.

### The surface velocity

We erected four ablation stakes near the GPR survey area (Fig. 1c) on August 3, 2008 and measured the amount of ablation on September 14, 2009. The melted depth and ice velocity were measured during the past year. Due to the complexity of the surface terrain and melt, the observed melt depth and ice velocity probably differed from the actual melt depth and velocity at the same absolute position, but we assumed that the difference was relatively small.

### Glacial surface temperature

Because there was no weather station on H8, we used the annual mean temperature data from 1958 to 2006 recorded at the Urumqi weather station near H8. According to the principle of air temperature decrease by 0.6 °C for every 100 m of elevation gradient above sea level, we calculated that the average temperature of the Urumqi weather station near H8 was −4.1 °C and the temperature at the position of the terminus of H8 was −5.9 °C, while the temperature of the highest elevation in the GPR survey was −9.5 °C. The mean temperature trend rate of the 14 weather stations around the Tianshan Mountains was 0.34 °C (10a)^−1^ ^39^. This warming trend was close to the RCP 4.5 result.

### Grid settings

**Figure 3:**
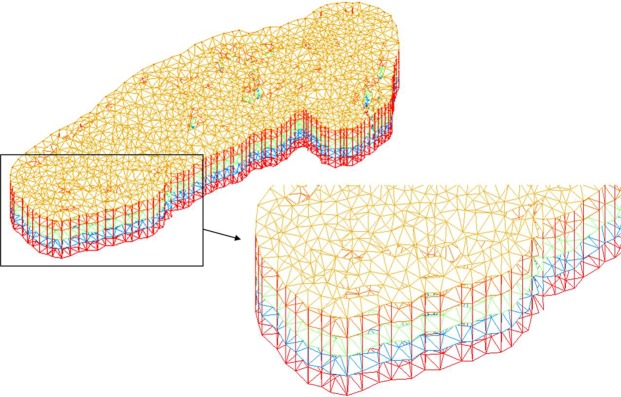
Finite element space grid setting.

The grid was divided into five layers in the simulated area, each of which used the same number of nodes to simulate the related dynamics characteristic (Fig. 3). The grid matches the distribution of the area of each layer to compress and extend the grid appropriately. To test the sensitivity of the grid resolution, a grid independence test was performed by systematically increasing the number of grid nodes and comparing the results. After comprehensively considering the convergence, accuracy and efficiency of the simulation, five grid systems were adopted. We used a regular grid with respective grid sizes of 100, 80, 60, 40, and 20 m. The extents of the resulting disturbance and rationality were regarded as the standards for evaluating the grid.

**Figure 4:**
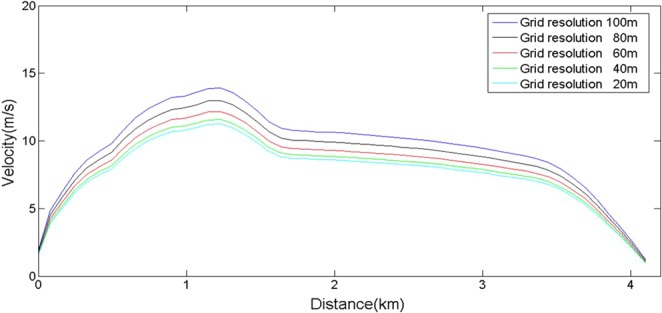
Result of grid independence test.

The tests showed that suitable simulation results could be achieved in a 40 m resolution grid (Fig. 4). The disturbances of both velocity and computational efficiency were suitable. We found that matching between the grid and terrain was very important. If the terrain resolution was denser than the model grid, this would lead to some erroneous results as well as increase the tedious calculation time. Therefore, we matched the terrain resolution with the grid.

### Sensitivity analysis

**Figure 5:**
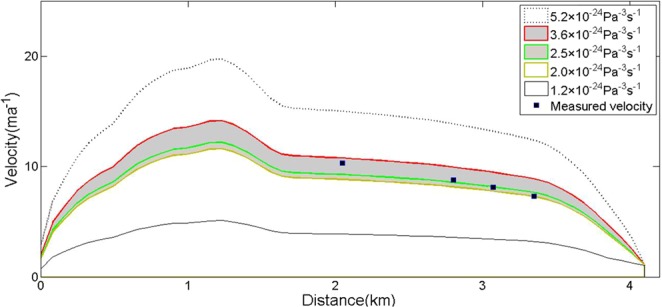
Measured and simulated glacial surface velocity along the main streamline. The simulated surface velocity represented different values of the flow - law coefficient *A* without basal flow.

Although we used a complex model and a great number of parameters in our study, there were no other verification data except the ablation stake and GPR thickness data obtained from field surveys. Therefore, we used the general or default value of the previously simulated ice flow for most parameters. For example, the value of Glen parameter *n* was 3, and the range of initial equivalent strain rate *ε* was from 1.2 × 10^−4^ to 2.2 × 10^−4^; the value of the universal gas constant *R* was 8.314 Jmol^−1^ K^−1^, the ice thermal conductivity value *k* was 1.8, and the range of flow - law coefficient *A* was generally from 1.0 to 3.4 × 10^−24^ Pa^−3^ s^−1^ ^40^. We tested the change in surface velocity with parameter *A* (Fig. 5).

The disturbance that truly affected the simulation results mainly came from the inputted ice thickness and surface temperature boundary conditions. We inserted the overall thickness distribution and surface air temperature from the measured data. The model assumed that the velocity and temperature inside the glacier were continuously distributed, but in fact, the distributions of thickness and surface temperature were uneven in three-dimensional spaces. However, we could still obtain results close to the measured data velocity, and the ice temperature results were also in compliance with the actual ice temperature distribution, as shown in the simulation results of several kinetic parameters. The velocity, stress and ice temperature were mainly dependent on the ice thickness and atmospheric temperature.

Precise knowledge of the basal conditions and implementation of a good sliding law are crucial for modelling. There are three types of sliding conditions in the Elmer/Ice model. The first two methods assume that a linear relationship exists between the bottom sliding velocity and shear stress. The third method gives relatively complex descriptions of the bottom sliding velocity and shear stress^41^. In this study, we used these three types of sliding laws. Then, the separate sliding velocity range of 4 ms^−1^ to 6 ms^−1^ was obtained at the bottom. Subsequently, we compared and analysed the temperate ice zone in the GPR data and thought that a sliding velocity of over 4 ms^−1^ at the bottom was impossible. Therefore, we set the freezing state at the bottom. It should be emphasized that the simulation of basal sliding velocity was calculated as a separate module in the Elmer/ice model, which had no effect on the glacial average velocity.

## Results

### The velocity simulation result

**Figure 6:**
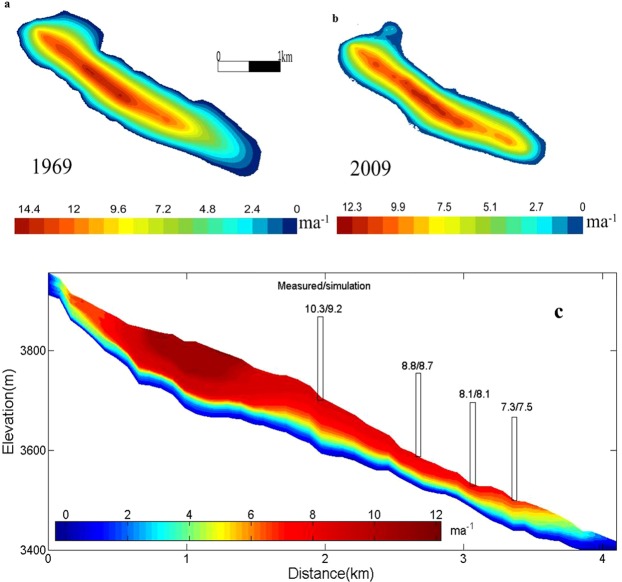
The simulated surface velocity. (**a**) The simulated surface velocity in 1969; (**b**) the simulated surface velocity in 2009; (**c**) the velocity distribution in the mainstream line in 2009.

Figures 6a,b present the velocity distributions from two thickness data based on the Full-Stokes code Elmer. The maximum velocities obtained by the thicknesses of the two stages were 14 and 12 ma^−1^, respectively. Figures 6a,b also show that the maximum velocity of the glacier decreased with decreasing ice thickness. This result was consistent with the relationship between the velocity and thickness in Eq. 2 and was consistent with trends in the velocities of some large glaciers^42^; that is, the effect of air temperature on the velocity of large glaciers is small. However, the variation in the velocity of the main flow line was more inhomogeneous in 2009 than in 1969, indicating that the surface terrain may fluctuate greatly. The meteorological station observations show that there was an air temperature rise of 1.36 °C near the region, and the warming rate was highest during 1969 to 2009. The effect of the reduced thickness on the velocity was compensated for by increased ice rheology.

According to the comparisons between topographic maps in 1969 and GPR measurements in 2009, the variation in thickness implied an average reduction of 1.13 m per year. The maximum thickness in the comparison was reduced by one-sixth, but the decreasing rate of maximum velocity was less than one-sixth, indicating an increase in the local velocity of the glacier. From Fig. 5c, we found that the velocity in the simulated main flow line profile coincided well with the measured data. Therefore, the simulation results in our study generally reflect the actual situation.

### The temperature simulation result

**Figure 7:**
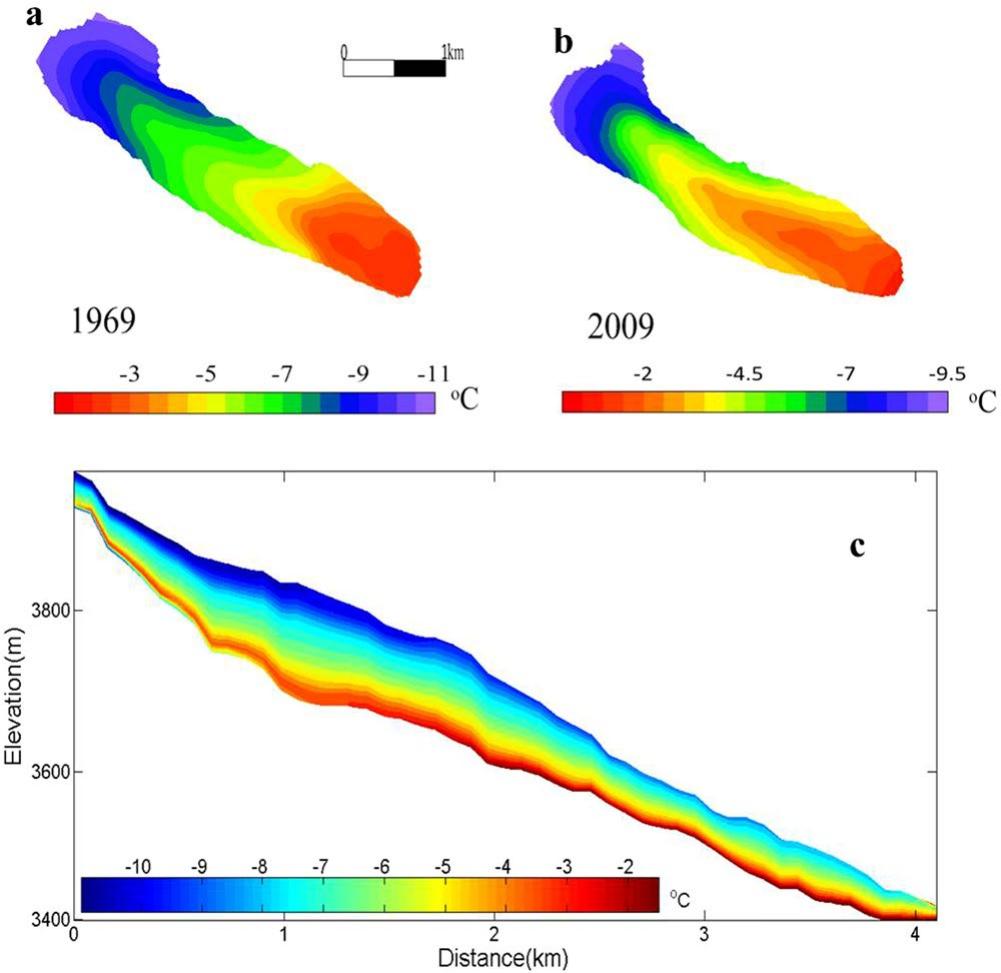
The simulated temperature. (**a**) The simulated temperature at the bottom in 1969; (**b**) the simulated temperature at the bottom in 2009; (**c**) the ice temperature distribution in the mainstream line in 2009.

The ice temperature is an important indicator of glacial change, especially the ice temperature at the bottom, which has an important effect on bottom sliding and ablation^43,44^. Figure 7 presents the distribution of ice temperature at the bottom for 1969 and 2009 obtained from the diagnostic simulation. There was a certain increase in the ice temperature at the bottom from 1969 to 2009. An analysis of the temperature trend indicated that the annual average temperature increased by approximately 0.18 °C (10a) from 1959 to 2007^45^.

Under the same geothermal conditions, the air temperature is the main cause of the ice temperature rise. The higher pressure can lead to additional frictional heat and latent heat release, which can accelerate ice ablation at the bottom^46,47^.

Figure 7c shows the two-dimensional distribution of ice temperature, which is similar to the findings of Wu^48^ and Zwinger^49^. Although ice core temperature data were not obtained in our study, the vertical distribution of the simulated ice temperature was close to the measured ice temperature of the Miaoergou Glacier in the eastern Tianshan Mountains^50^. The ice temperature range of Miaoergou was from −2 °C to −8 °C, while the ice temperature range in this study was from −0.5 °C to −0 °C.

### The deviation stress simulation result

**Figure 8:**
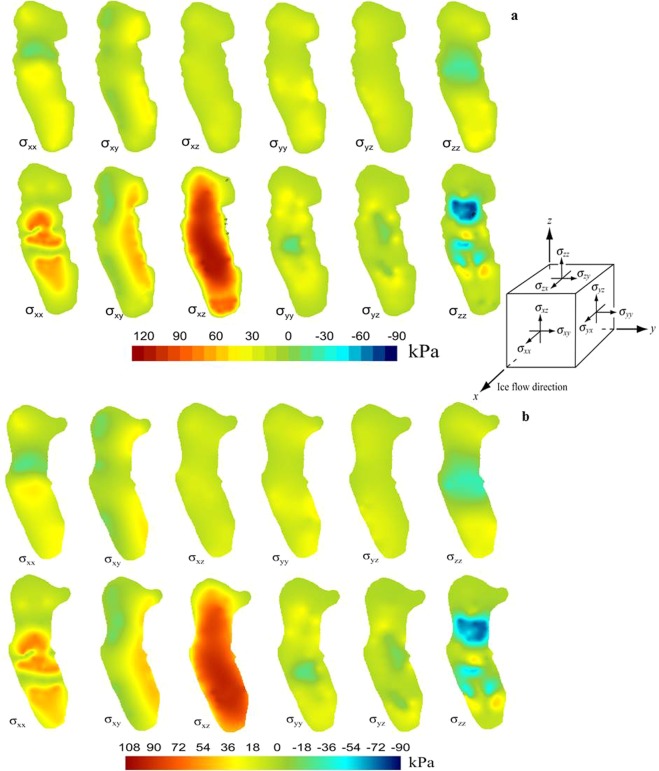
The simulated deviation stresses. (**a**) The simulated deviation stresses on the surface and at the bottom in different directions in 1969; (**b**) the simulated deviation stresses on the surface and at the bottom in different directions in 2009.

Generally, ice is considered to be a nonnewtonian fluid with high viscosity, i.e., the variation in shear stress with shear strain rate is nonlinear. The linear relationship between these factors also depends on the viscoplasticity; for example, large ice viscosity in winter is more likely to cause ice deformation and development of crevasses^51,52^. To analyse the variation in stress caused by the variation in ice thickness, we simulated the change in shear stress in different directions and found that the deviation stress played an important role in the creep process of glaciers. Figure 8 shows the deviation stress distribution on the surface and bottom of the two-time node glacier. Overall, as a result of the direct effect of the atmosphere temperature, there was a subtle change in the deviation stress spatial distribution. Although the complex forces were superimposed in different directions, the deviation stress of the surface was obviously smaller than that of the bottom. However, the deviation stress on the bottom was not only affected by the gravity of the whole glacier but also by the combined effects of rocks or melting water, so the stress may change greatly over a long period of time^53^.

The warm and cold colours represent the compressive stress and tensile stress in Fig. 8, respectively. By comparing the surface stresses under conditions of different thicknesses and temperatures, we found that the stress on the surface dispersed in all directions in 2009 and the compressive stress downstream showed an overall increasing trend, with an especially distinct expansion at the terminus. This result may be related to the reduction in glacial scale and thickness because the large scale and thickness could prevent ice flow from upstream, resulting in local abnormal pressure. It could be concluded that theoretically, the deviation stress *σ*_*zz*_ on the surface should be small due to the minimum gravity, but the deviation stress *σ*_*zz*_ on the surface showed a local increasing trend. Under the influence of a steep slope, there was a depressed ice flow stretching across the surface ice. The deviation stress *σ*_*xx*_ on the surface is the deviation stress produced by the gravity component, which determines the velocity on the surface. Both results showed that the velocity near the corner of the ice flow was higher than the average velocity around it, indicating that the topography greatly promoted the stress variation, so the increase in compressive stress in the upstream area may be related to ice flow narrowing.

By comparing the deviation stresses *σ*_*xy*_, *σ*_*yz*_ and *σ*_*yy*_ in three directions, we found that the distribution of deviation stresses *σ*_*xy*_ and *σ*_*yy*_ changed slightly from the simulation results, indicating that the topography and thickness had little effect on the lateral force balance. The intensity of the deviation stress on the surface was not pronounced, but that at the bottom was the opposite. Because of the influence of gravity, the friction force and twisting deformation force in bedrock, the stress varied obviously with thickness, and the maximum compression and tension forces of the ice body appeared at the bottom. The results revealed that the maximum compressive stress decreased by 12 kPa, but the tensile force hardly changed, indicating that the thickness changes affected the distribution of the extrusion force. However, the tensile force was mainly caused by the difference in ice velocity from 1969 to 2009. This result meant that the deviation stress shared certain gravity in both the horizontal and vertical directions and maintained a relative balance, which ensured that the stresses on the surface and bottom remained within certain ranges. Moreover, the hardness and melting point of ice also made the ice around the bedrock unable to withstand large deviation stress. When the stress exceeded the capacity of the ice to withstand pressure, the ice could break or melt to release the stress accumulation.

In Fig. 8, the deviation stresses *σ*_*xy*_ and *σ*_*xz*_ at the bottom changed greatly, where the former represented the gravity component of the ice flow direction, and the latter represented the additional force caused by the gravity component in the terrain of the ice-rock interface. From the simulation results, we found that the ice at the bottom was the maximum force position, which was most prone to deformation, distortion and melting, and the reduction in the pressure melting point could increase this trend.

The results of the stress simulation showed that the glacier was a complex fluid, and the reason for the spatial stress anomaly could be explained by the different spatial stress caused by climate change. The acceleration of ice motion was proportional to the product of strain rate. In particular, the protrusion or depression of bedrock would increase the ice flow distortion stress and reduce the ice movement velocity^36^. If there was closed melting water in the ice bed position, the sliding speed and stretch effect would both increase and promote ice fracture. We could see from the changes in the whole force that the force at the bottom was key to maintaining the overall stress balance, and glaciers that melt and collapse in bedrock would more easily destroy the overall force balance and increase the glacial ablation change rate.

### The verification of stress

**Figure 9:**
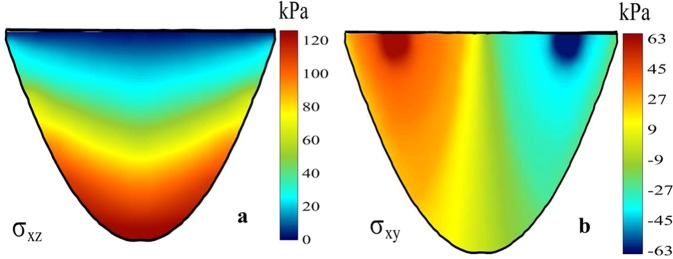
The simulation englacial stresses. (**a**) *σ*_*xz*_ and (**b**) *σ*_*xy*_^54^.

To verify the stress simulation, we tried to compare these results with similar Full-Stokes studies. The previous study also used the Full-Stokes method to simulate several deflection stress results. For example, Surendra simulated the stress distribution on a glacial cross section^54^. Surendra’s simulation deviation stresses *σ*_*xz*_ and *σ*_*xy*_ are shown in Fig. 9. Compared with our simulations of these stresses in the corresponding direction, we found that the distributions of stresses were consistent with his study, both on the surface and at the bottom of the glacier. The magnitude of stress was related to the ice thickness, so we could only verify our results by the distribution of stress.

### The prognostic simulation result

In this study, we replaced the change in mass balance in the future with the variation in average thickness from 1969 to 2009. Changes in glacial thickness at different altitudes in the future were expressed by the following formula:

13 $$ELA=EL{A}_{2009}+\alpha \Delta T(year)(year > 2009)$$

where Δ*T* is the net change in temperature relative to the *k*th year and *α* is the sensitivity of elevation (ELA) to temperature changes.

**Figure 10:**
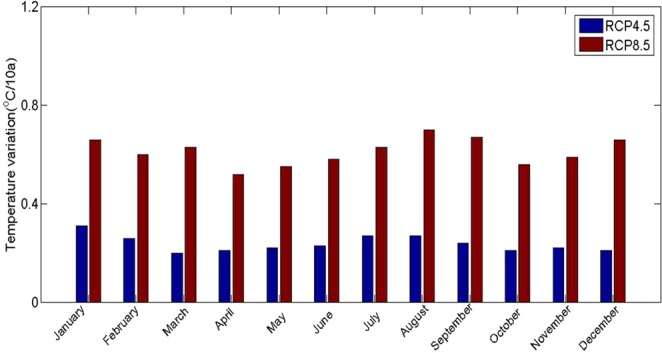
The monthly slope of the temperature under the RCP 4.5 and RCP 8.5 scenarios.

Although the thickness data can be calculated from the corresponding relationship between temperature and thickness, the long-term variation in glacial thickness includes not only glacial ablation but also changes in surface elevation caused by ice flow movement. To reflect the changes in ablation in different regions, we distributed the total thickness changes to different altitudes according to the temperature gradient, and the total thickness changes in different gradients were equal to the average thickness changes during two periods. The variation in glacial thickness (*α* value) at different altitudes from 1969 to 2009 was obtained by the thickness subtraction calculation. The calculation process and results were as follows: −1.25 ma^−1^, −1.18 ma^−1^, −1.05 ma^−1^, −0.92 ma^−1^, and −0.71 ma^−1^ were obtained from 3394 minus 3594, 3594 minus 3694, 3694 minus 3794, 3794 minus 3894, and 3894 minus 4000 m.a.s.l., respectively. In this case, the initial surface velocity referred to the simulated velocity in this study. In terms of the relationship between the thickness variation and atmospheric temperature, from 1969 to 2009, when the atmosphere temperature increased by 1 °C, the average thickness decreased by 36.03 m. The variation in thickness was distributed in different elevation zones in the same proportion. Simultaneously, we used the RCP 4.8 and RCP 8.5 climate models to obtain the future atmospheric temperature changes (Fig. 10).

**Figure 11:**
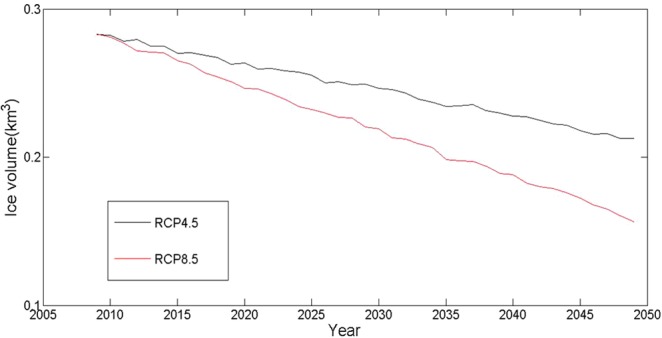
Glacial volume changes from 2009 to 2049 based on the RCP 4.5 and RCP 8.5 climate modes.

Figure 11 displays the volume changes in H8 over the next 40 years based on two climate models. Clearly, the atmospheric temperature was the most important driving factor of glacial ablation. Under the two climate models, the average annual temperature would increase by 0.612 °C 10a^−1^ and 0.237 °C 10a^−1^, respectively, and the variation in average annual temperature would increase by nearly three times. However, the volume change every 10 years caused by the RCP 4.5 and RCP 8.5 models would be 0.14 km3 and 0.232 km3, respectively, and the difference between the reductions in volume would be less than two times. This phenomenon indicated that the response of glaciers to temperature had a threshold and a nonlinear change. When the ice temperature approached the threshold, the movement and ablation of the glaciers would accelerate. On the other hand, the response of glaciers to climate changes was also proven to be lagging.

**Figure 12:**
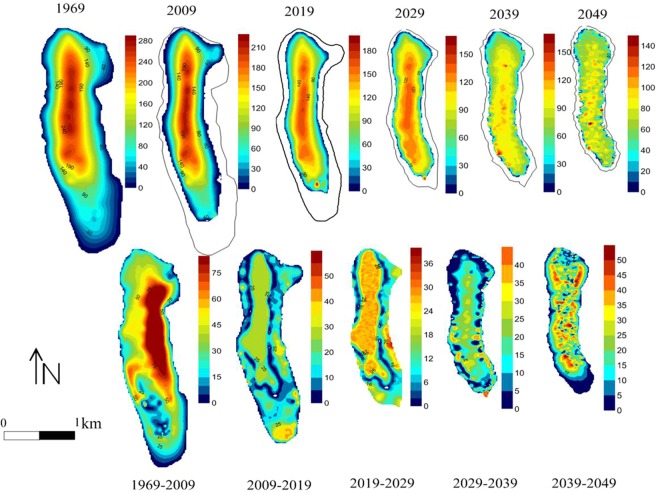
Thickness distribution of glaciers from 2009 to 2049 and variation in thickness at an interval of 10 years.

Figure 12 shows the variation in the area and thickness of glaciers under the RCP 4.5 model. From 2009 to 2029, the variation in thickness would be steady, and the irregular ablation after 2029 would lead to uneven spatial thickness change. During this period, the maximum thickness change in the glaciers was obvious, far exceeding the maximum average thickness change rate from 1969 to 2009. The simulation results showed that the variation in thickness in the location of the main flow line began to intensify from 2009 to 2019, indicating that the ice flow would experience a diffusion process. Similar changes would be observed from 2019 to 2029, but the ice thickness in the terminus did not change significantly. During the period from 2029 to 2039, although the main variation in thickness would originate from the mainstream line, irregular melting occurred in different locations on the surface. This kind of melting change strengthened from 2039 to 2049. Then, distinctly uneven ice surface topography formed, and the topographic relief gap reached 20 m. Subsequently, the surface velocity was obviously affected by the local glacial topography. The spatial difference in ice velocity led to the generation of interstitial compressive flow on the surface. With the decrease in ice thickness, the oblique downward component of gravity decreased obviously, and the ice flow velocity was largely controlled by the topography. In addition, the differential effects of surface moraines, ice surface temperature, radiation and other factors increased the nonuniformity of glacial stress and aggravated the irregular variation in thickness.

### The glacial variation verification

**Figure 13:**
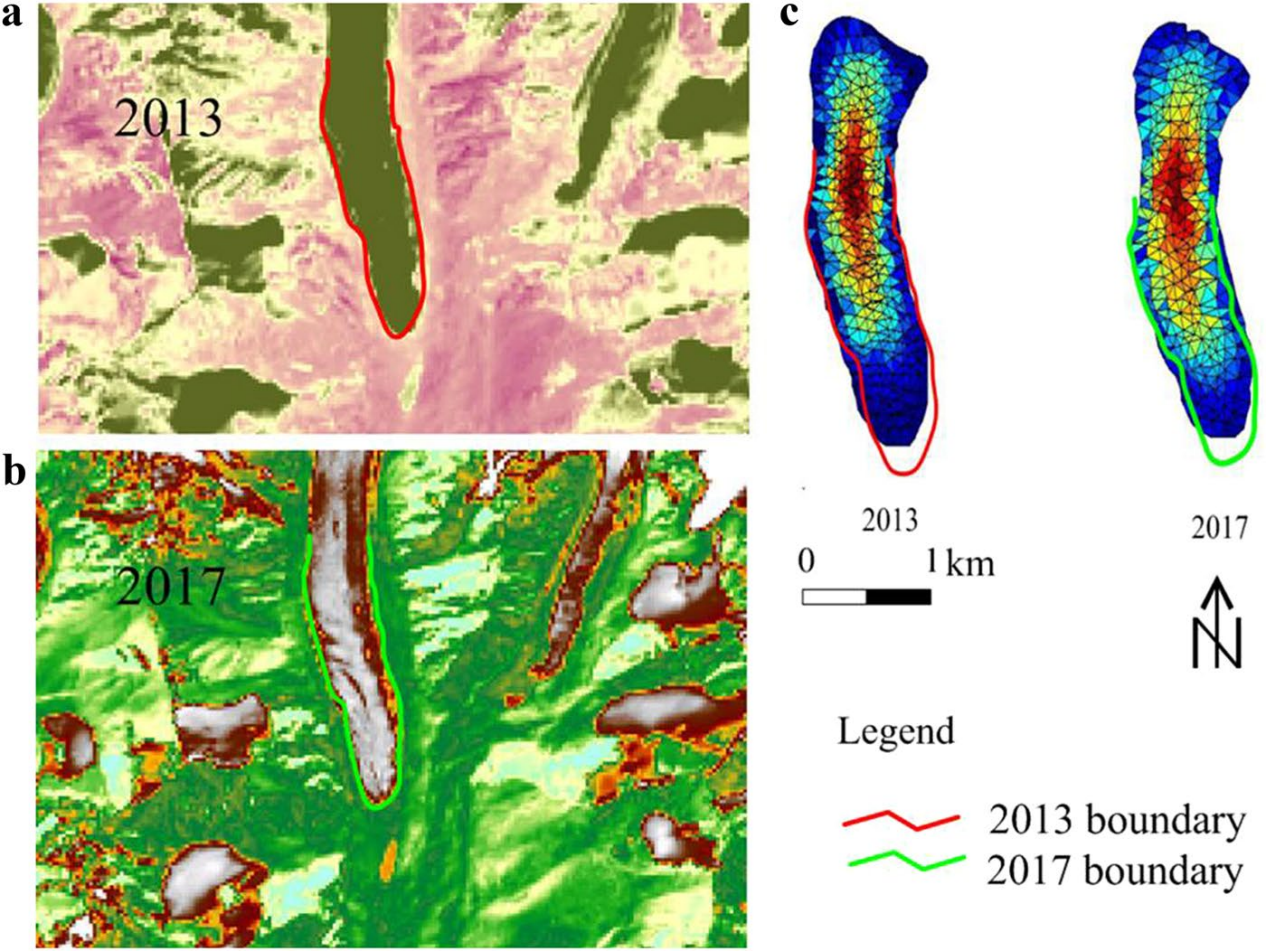
The overall of comparison of the simulation results with Landsat images (https://glovis.usgs.gov/). (**a**) Landsat image in 2013 and the map was plotted using ArcGIS 10.2 (http://www.esri.com/). (**b**) Landsat image in 2017. (**c**) Comparison of the simulation results with Landsat images.

The combination of movement speed and ablation acted on the variation in glaciers. In Fig. 11, the glacial thickness distribution from 2009 to 2049 and thickness variation were simulated at intervals of 10 years. To verify the prognostic simulations, we compared our simulated glacial profile with Landsat images in 2013 and 2017 (Fig. 13). The variations in the glaciers in the remote image were slower than those in the simulation. We analysed the reason why the simulation variations were smaller than the actual changes. The main reason was that the ice flow supply upstream was not considered in the model, and the ice supply upstream in the simulation mainly came from snowfall. Simultaneously, the alternation of the four seasons and change in the equilibrium line would also affect the glacial rheological state. Second, the resolution of the model did not fully consider the impact of a specific terrain slope on the ablation and rheological state, which can cause some errors. In the future, improvements in the model’s temporal and spatial resolutions are necessary to reduce these errors. In addition, we found that the small changes in the boundaries did not indicate small impacts of climate or ablation amount. The variations in internal structure were invisible, and the ablation and collapse of the ice bodies inside the glaciers were ignored in the model. Therefore, it is necessary to increase the future hydrological effect model to fully describe the glacial response to climate change.

The mass balance and stress during glacial changes need time to rebuild, and the change in the area of the H8 glacier shows an alternating change process, which also explains the delay in the glacier’s response to climate change.

## Discussion

### The effects of thickness and stress on ice flow velocity

The Full-Stokes model describes the dominant physics of the system, where the gravitational driving stress is exactly balanced by the internal and basal shear stresses^34,48,55^. In this study, the ice velocity corresponding to the simulation stress could better match the actual observed results, which indicated that the models and parameters we chose are suitable for both diagnostic and prognostic simulations. According to Eq. 2, the component of gravity controlled the stress in different ice flow directions. In other words, the topography at the bottom, the gradient of the local ice flow and the flux of the ice flow all contributed to the stress balance^34,56^.

However, from 1969 to 2009, the average ice thickness decreased by approximately 15 percent, but the stress variation magnitude was not significant. This finding implies that glaciers can change the pressure distribution through crushing and creep, and the bearing capacity of ice to stress is also maintained in a certain range. For example, when the stress at the bottom is large, the reduction in the pressure melting point will increase ablation, collapse and basal sliding speed. When the stress is small, the ice body will move with the whole ice flow, and the internal melting of the glacier is mainly infiltrated by outside temperature and melt water. In other words, glaciers release and accumulate this stress by changing the speed of movement.

Generally, the variation in observation velocity reflects the extent of internal stress variation. First, the decrease in thickness means that the horizontal driving force produced by the gravity of the glaciers is reduced^57^, and therefore, the motion velocity is also reduced. If the velocity of glacial migration increases with decreasing ice thickness, this indicates that glacial viscoplasticity is significantly affected by climate. Some remote sensing observations in low latitude areas show that there is only a small area increase in the local glaciers^5^, which is likely to be a potential precursor to accelerated ablation. In the case of the Columbia Glacier, Van der Veen empirically linked terminus retreat with terminal thickness and concluded that terminal retreat was likely triggered when the terminal thickness was less than 50 m above the (local) flotation thickness^58,59^. Forward ice flow is also the reason for the rapid regression of small scale glaciers^60,61^.

Moreover, the gravity component of the surface ice flow could be increased by the increasing surface gradient, while the local topographic changes could only change the velocity of the protruding part but had little effect on the velocity of the deep ice layer. Simultaneously, the fluctuation in surface topography increases under the influence of further erosion by melt water, and finally, the ice surface topography shows a serac morphology. The simulation results show the influence of stress on the surface topography.

### Effect of atmospheric temperature on ice flow velocity

Because the geothermal energy at the bottom was determined, there was no disturbance to the simulation results. The fluctuation in the simulation results mainly came from the influence of atmospheric temperature. Equation 4 reveals an obvious functional relationship between creep and ice temperature. The results of a previous study suggest that small variations in ice flow surface velocity are strongly correlated with air temperature^52,62^. In some main glacial branches, the ice velocity on the surface reaches a peak from late winter to midsummer and reaches the minimum between late summer and fall. The near-front velocity of the ice reached up to 14 mday^−1^ in May and the lowest speed of 1 mday^−1^ in October^63^. Although there also exists a close relationship between the basal water pressure and surface velocity^64^, the contribution of temperature to the increase in velocity accounts for a considerable proportion. Figure 6 shows that the maximum surface velocity only changed by 2.1 ma^−1^ from 1969 to 2009, while the maximum thickness decreased from 280 m to 210 m, indicating that there were other factors that could compensate for the acceleration loss caused by the gravity component. Although the velocity was the result of the combined interaction of thickness and ice temperature, the effect of the two on the velocity was the opposite. Thus, the flow velocity changed little because of the counteracting effect with the decrease in ice thickness and the increase in ice temperature at the same time. In addition, Howat *et al*. inferred that terminal retreat can also contribute to surface flow by weakening the resistance stress^65^, mainly by improving the ice flow velocity downstream. Compared to the results of the XD Glacier study in the Qinghai-Tibet Plateau using the Elmer/ice model^48^, according to the geothermal catalogue, the same geothermal value is used at the bottom, but only the difference in surface temperature leads to the maximum ice velocity of H8 being nearly five times greater than that of XD.

To study the influence of ice temperature on ice flow movement, we selected two ice profiles of 180 m and 80 m in 2009 to simulate the sensitivity of ice flow velocity to temperature, which was mainly to simulate the influence of atmospheric temperature on the velocity when the temperature increases or decreases by 1 °C.

**Figure 14:**
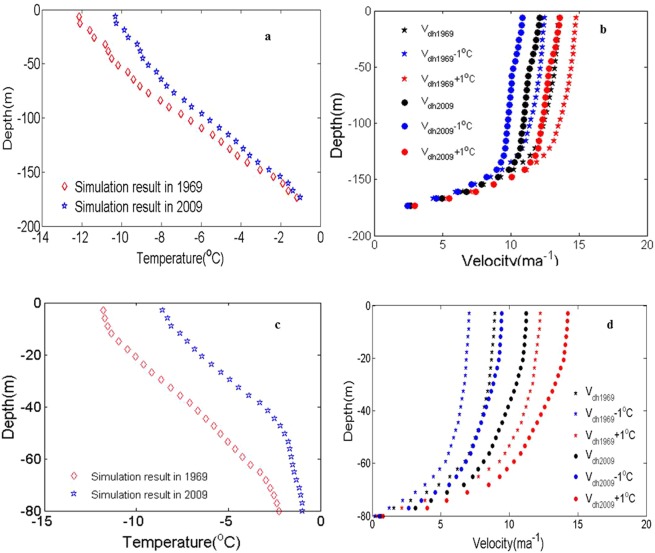
The simulated ice temperature and velocity under different ice thicknesses. (**a**) The simulated ice temperature in 1969 and 2009 under the condition of ice thickness of 200 m and atmospheric temperature; (**b**) the variation in velocity under the condition of ice thickness of 200 m and a rise in atmospheric temperature by 1 °C in 1969 and 2009; (**c**) the simulated ice temperature in 1969 and 2009 under the condition of ice thickness of 80 m and atmospheric temperature; (**d**) the variation in velocity under the condition of ice thickness of 80 m and a rise in atmospheric temperature by 1 °C in 1969 and 2009.

The final steady-state simulation results are shown in Fig. 14a,c. These results indicate that the surface temperature of the glaciers could affect the deep ice temperature. As long as the surface temperature lasts for a certain period of time, it can affect deep ice temperature changes. Simultaneously, a smaller change in air temperature can lead to a greater change in the overall velocity profile.

Due to the low resolution of the model, the surface ice did not show obvious characteristics of the active layer temperature (Fig. 14a,c), but the slow change in surface ice temperature proved that the variation in ice temperature may be reversible. Figure 14b,d show the change in velocity caused by a temperature rise or drop of 1 °C in the two profiles under the current temperature conditions. The results indicated that the higher the ice temperature was, the greater the velocity increased under the same temperature change. In addition, previous researchers have also observed and simulated the ice flow velocity in different seasons, which proves that higher ice temperature has a greater impact on velocity improvement.

### Effects of stress on englacial and subglacial structures

As far as we know, stress can change the englacial structure, distribution of cracks and drainage systems^66,67^. The occurrence of englacial drainages initiated by hydrofracturing in diverse glaciological regimes suggests that it is a very widespread process. As long as the high meltwater supply coincides with ice subjected to sufficiently large tensile stresses, drainage will occur from the surface to the bed^66,68^. Figure 7 demonstrates that the maximum stress was located in bedrock, and the stress changed significantly over time because the fluctuant amplitude of the rough interface made the compression force and tension force distributions at the bottom more complex, which suggested that the basal topography played an important role in controlling the dynamic response^69–71^.

Due to the effect of changes in glacial morphology on stress balance, especially by both upstream compressive force and downstream tensile force, the different viscous forces of the ice body lead to complex interleaving of transverse stress and longitudinal stress. These irregular forces lead to more englacial crevasses and channels, thus accelerating the melting and retreat of glaciers.

The basal stress is an important underlying factor for scaling glacial erosion^34^. When ice flow at the bottom suffers differential stress from different directions, the ice is more likely to break and distort, and ablation intensity increases^72,73^. Simultaneously, local heterogeneity, such as the presence of cavities, may concentrate stress at much higher values^34^. The erosion rate is commonly assumed to be on a scale of base sliding velocity or ice discharge^74,75^. With the development of englacial melting holes, supraglacial melt water is able to rapidly reach the bed at elevations below equilibrium line via connections among moulins and crevasses^76^. The locally closed melt water promotes basal sliding, which can also result in more basal melt through frictional heating^77^. In our study, the simulated stress at the bottom in 2009 was greater than that in 1969, which may be related to the elastic - viscous deformation of ice caused by the temperature increase set in the models.

The simulation results of the bottom stress revealed that the maximum stress distribution was maintained on the bedrock for most of the time, especially the deviation stress in the gravity direction. Thus, the ice flow at the bottom is simultaneously affected by two factors: tectonic forcing^78,79^ and transient climate forcing^80^. Therefore, the ice flow at the bottom is the most prone to collapse in vulnerable locations. With the change in glacial morphology, the deformation or collapse will be prompted by the changes in stress in different directions at the location where the stress is greater. More importantly, a climatic response will occur first. Thus, our research provides a good method for judging vulnerable glacial locations and predicting future trends under different climatic conditions.

### Enlightenment to glacial change in the Tianshan area

Previous studies have shown that most glaciers in the Tianshan Mountains present generally negative trends over the past half century, in which the increase in the mean temperature in summer is the main cause of glacial shrinkage^81,82^. However, there are significant differences of individual glaciers in response to climate changes^82,83^, as our study in H8 reflects this characteristic. Our simulation results showed that with the decrease in glacial thickness, all of the stress decreases. First, the decrease in ice thickness will reduce the ice velocity and ice supply from upstream to downstream. The high summer temperatures can accelerate ice velocity. The high summer temperatures can also increase ice flux movement and promote glacial area in the low and middle altitudes. Moreover, slowing of the average glacial velocity and increased thinning of glacial thickness jointly promote the retreat of glaciers in the Tianshan Mountains, especially small scale glaciers with slow velocity and shrinkage rate, which are more likely to show accelerated retreat in the absence of ice supply from upstream. Some studies have shown that there is an increasing trend in glacial area under the background of climate warming^5^. The viscoplastic deformation of glaciers under the influence of high temperature may lead to an increase in glacial area. Our study also indicated that an increase in area may not mean an increase in mass balance but may indicate an increase in ice velocity caused by warming.

## Conclusion

In this study, we simulated the ice velocity, temperature and deviation stress under glacial thickness conditions in 1969 and 2009 and analysed the influence of climate and thickness change on glaciers due to stress variations in the future. The diagnostic simulation showed that the ice flow velocity was mainly influenced by the combined action of the gravity component and ice viscous force, and the homogeneity of the regional force was the reason for the complexity in glacial movement. The atmospheric temperature variations led to ablation and caused differences in ice temperatures to shape the differential forces of the ice body, which resulted in a complex balance of the subglacial and englacial compressive and tensile stresses. With the decrease in thickness and surface velocity, coupled with the spatial difference in ablation and difference in surface velocity, the difference in mass balance was magnified. The formation of icebergs implies the intensification of this trend. With the change in stress and ice flow structure caused by ablation, the location of ice flow at the bottom will more likely facilitate the occurrence of various hydrological processes. Sliding-induced ablation of the basement and strain softening of basal ice will promote the acceleration of ice flow. The prognostic simulation showed the variation tendency of thickness and area. The results suggested that the abnormal surface ablation caused by atmospheric temperature and ice thickness is related to the variation in surface velocity and ice flow viscoplasticity.

The entire study shows that the difference in regional force caused by accumulated ablation and ice flow is the cause of the complicated glacial movement. The short-term area increase may not indicate an increase in mass balance, but it may be an increase in ice velocity caused by warming. The Elmer/Ice model is suitable for the simulation of glacial dynamics in areas of complex terrain and morphology. This model has good application prospects in cryospheric research. Combined with other cryospheric studies, the application of the Elmer/Ice model can explain more glacial motion mechanisms.
